# CRISPR/Cas9-Mediated Generation of Pathogen-Resistant Tomato against *Tomato Yellow Leaf Curl Virus* and Powdery Mildew

**DOI:** 10.3390/ijms22041878

**Published:** 2021-02-13

**Authors:** Dibyajyoti Pramanik, Rahul Mahadev Shelake, Jiyeon Park, Mi Jung Kim, Indeok Hwang, Younghoon Park, Jae-Yean Kim

**Affiliations:** 1 Division of Applied Life Science (BK21 FOUR Program), Plant Molecular Biology and Biotechnology Research Center, Gyeongsang National University, Jinju 660-701, Korea; dpinbiotech@gmail.com (D.P.); ecastle109@gmail.com (M.J.K.); 2 Department of Horticultural Bioscience, Pusan National University, Miryang 50463, Korea; o_omad@naver.com; 3 R&D Center, Bunongseed Co., Ltd., Gimje 54324, Korea; usename@hanmail.net

**Keywords:** CRISPR, genome editing, tomato, TYLCV, powdery mildew, trait improvement

## Abstract

Tomato is one of the major vegetable crops consumed worldwide. *Tomato yellow leaf curl virus* (TYLCV) and fungal *Oidium* sp. are devastating pathogens causing yellow leaf curl disease and powdery mildew. Such viral and fungal pathogens reduce tomato crop yields and cause substantial economic losses every year. Several commercial tomato varieties include *Ty-5* (*SlPelo*) and *Mildew resistance locus o 1* (*SlMlo1*) locus that carries the susceptibility (*S*-gene) factors for TYLCV and powdery mildew, respectively. The clustered regularly interspaced short palindromic repeats (CRISPR)/CRISPR-associated protein (Cas) is a valuable genome editing tool to develop disease-resistant crop varieties. In this regard, targeting susceptibility factors encoded by the host plant genome instead of the viral genome is a promising approach to achieve pathogen resistance without the need for stable inheritance of CRISPR components. In this study, the CRISPR/Cas9 system was employed to target the *SlPelo* and *SlMlo1* for trait introgression in elite tomato cultivar BN-86 to confer host-mediated immunity against pathogens. *SlPelo*-knockout lines were successfully generated, carrying the biallelic indel mutations. The pathogen resistance assays in *SlPelo* mutant lines confirmed the suppressed accumulation of TYLCV and restricted the spread to non-inoculated plant parts. Generated knockout lines for the *SlMlo1* showed complete resistance to powdery mildew fungus. Overall, our results demonstrate the efficiency of the CRISPR/Cas9 system to introduce targeted mutagenesis for the rapid development of pathogen-resistant varieties in tomato.

## 1. Introduction

Tomato (*Solanum lycopersicum*) is one of the agronomically important food crops consumed worldwide. A recent analysis of the tomato market by Food and Agriculture Organization Statistics [1] showed a total of 188 million tons globally that generated USD 190.4 billion in 2018. The tomato market is increasing every year. However, significant yield losses occur due to a wide range of vulnerabilities to different pathogens [2]. Among several tomato pathogens, *Tomato yellow leaf curl virus* (TYLCV), a circular single-stranded DNA (ssDNA) *Begomovirus* from the *Geminiviridae* family, is one of the most devastating viral pathogens affecting tomato cultivation, one that severely affects crop yield. TYLCV is transmitted by whiteflies (*Bemisia tabaci*) while feeding on plant phloem sap [3]. One recent report suggested that TYLCV can be seed-born because a viral particle can reside inside the seeds after infection and be transmitted to the next generation [4]. TYLCV exploits plant cell machinery to multiply its genome copy number and systemically spreads through the phloem to other plant parts to develop disease symptoms. Severe TYLCV infection can cause abnormal leaf morphology, including stunted growth, curling of margins, and yellowing. 

Several strategies have been employed to prevent TYLCV spread, including stringent quarantine rules, integrated pest management, conventional breeding, and genetic engineering [5]. The quantitative trait loci (QTL) linked with TYLCV resistance were recently identified, including *Ty-1*, *Ty-2*, *Ty-3*, *Ty-4*, *Ty-5*, and *Ty-6* in wild tomato varieties (summarized in [6]). The characterization of the *Ty-2* gene suggested its role in triggering a hypersensitive response in *Nicotiana benthamiana* upon TYLCV infection [7]. Notably, the *Ty-5* locus encodes the *SlPelo* gene that synthesizes a messenger RNA surveillance factor known as Pelota (PELO) [8]. The PELO protein is reported to play a critical role in ribosome recycling during protein synthesis, and *SlPelo* knockout mutants showed restricted TYLCV proliferation. 

Along with conventional methods, several transgenic strategies have been employed to tackle TYLCV transmission in recent times. Tomato plants ectopically expressing TYLCV gene segments such as capsid protein (*CP*) [9] or replication-associated protein (*Rep*) [10] provide resistance upon viral infection. In another report, model plant *N. benthamiana* overexpressing recombinant antibodies against Rep protein showed attenuated TYLCV symptoms [11,12]. RNA interference (RNAi)-mediated viral gene silencing approach was successfully applied to achieve immunity to TYLCV [13,14,15]. For instance, silencing of the tomato *SlPelo* gene revealed viral resistance [8]. Similarly, improving native plant immunity by overexpression of the plant immunity-related genes is a rational choice to enhance pathogen tolerance. For example, overexpression of *SlMAPK3* in tomato plants reduces TYLCV pathogenicity [16]. Overall, conventional breeding and transgenic approaches are promising to control TYLCV infection but have several limitations. Traditional breeding is a time-consuming process with a high risk of losing essential traits during domestication. The major disadvantage of transgenic methods is that the transgene needs to express continuously to enable pathogen-tolerant phenotype and, hence, be considered genetically modified organisms (GMOs) [17]. In the gene silencing approach, RNAi does not cause the complete gene silencing and needs the stable expression of RNAi components, and plant viruses have been reported to develop a counter-defense strategy by producing viral suppressors of RNA silencing [18]. Therefore, new strategies for the development of TYLCV-resistant tomato crop varieties are urgently needed. 

The clustered regularly interspaced short palindromic repeats (CRISPR)/CRISPR-associated protein (CRISPR/Cas) is a type II bacterial immune system engineered for genome editing purposes [19]. The CRISPR/Cas-based genome editing tools consist of two major components, single guide RNA (sgRNA) and Cas9 endonuclease. The sgRNA–Cas9 complex scans the genome for its target site with protospacer adjacent motif (PAM) and generates efficient DNA double-strand break (DSB). During the error-prone DNA repair process, mutations can be generated. The CRISPR/Cas technology has been successfully applied for generating designer crop varieties, including pathogen-resistant crops [17,20]. In recent times, either the pathogen genome or the host plant genes have been targeted by CRISPR/Cas to gain a disease-resistant phenotype. The efficacy of the CRISPR/Cas technology has been demonstrated to provide TYLCV resistance in *N. benthamiana* by targeting the viral genome [21,22,23]. Likewise, sgRNAs targeting the *CP* or/and *Rep* gene have shown reduced TYLCV accumulation in tomato crops [23]. Targeting the viral genome requires stable expression of the CRISPR/Cas system to gain viral immunity, therefore judged as GMOs.

In the present work, we demonstrated the application of *Streptococcus pyogenes* Cas9 (SpCas9)-mediated genome-editing technology to confer TYLCV resistance by targeting the *SlPelo* gene in the Korean elite tomato line, BN-86. The genome-edited *SlPelo*-knockout plants showed TYLCV resistance by restricting the viral DNA proliferation. Introgression of pathogen-resistant traits is highly desirable in commercial tomato cultivars. Therefore, we also targeted the *Mildew resistance locus o 1* (*SlMlo1*), a well-characterized susceptibility factor of fungal powdery mildew disease [24]. The CRISPR/Cas9-edited knockout *SlMlo1* tomato BN-86 line displayed complete powdery mildew fungal resistance. Overall, the present study demonstrates the potential of the CRISPR/Cas9 system to engineer elite tomato lines for multiple pathogen resistance.

## 2. Results

### 2.1. Target Selection and CRISPR/Cas9 Plasmid Vector Construction

**Figure 1:**
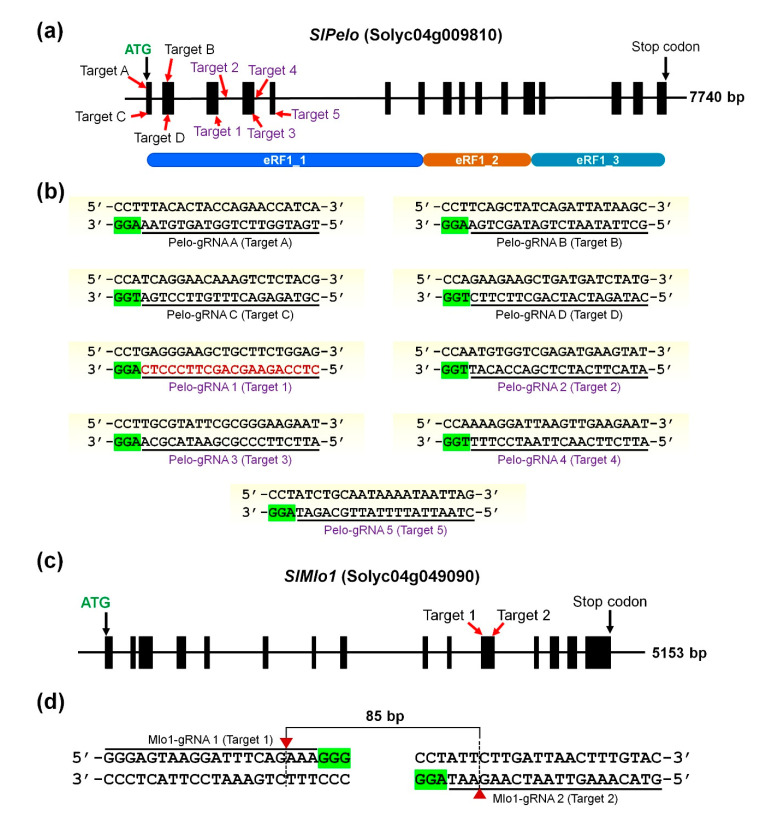
Schematic representation of the clustered regularly interspaced short palindromic repeats (CRISPR)/CRISPR-associated protein (CRISPR/Cas) target sites within *SlPelo* and *SlMlo1*. (**a**) Diagram showing the architecture of *SlPelo* locus. Black boxes indicate exons, and black lines indicate introns. The target sites are displayed with a red arrow. Three conserved eRF1 domains (eRF1_1, eRF1_2, and eRF1_3) predicted using InterProScan scanning of the Pelota (PELO) are parallelly positioned in the lower panel; (**b**) Nine guide RNAs (gRNAs) (underlined) along with their protospacer adjacent motif (PAM; highlighted in green) are summarized, corresponding to the *SlPelo* target sites as shown in panel (**a**); (**c**) Diagram showing *SlMlo1* architecture. Black boxes represent exons, and black lines indicate introns. The *SlMlo1* target sites are displayed with a red arrow; (**d**) The gRNA sequences (underlined) followed by protospacer adjacent motif (PAM; green). Red triangles with a dotted line showing the cleavage position of SpCas9.

In recent years, CRISPR/Cas9 technology showed immense potential in engineering the plant genomes for trait introgression in elite cultivars [25]. The PELO protein comprises 387 amino acids and retains three conserved eukaryotic translation termination factor 1 (eRF1) domains named eRF1_1, eRF1_2, and eRF1_3 (Figure 1a). The eRF1 domains of PELO reported playing a crucial function in ribosome recycling followed by the synthesis of proteins [26]. The RNAi-mediated *SlPelo* silencing exhibited TYLCV tolerance probably by slowing down or inhibiting ribosome recycling and decreasing protein synthesis in the infected cells [8]. Introduction of indel mutation in the eRF1_1-coding region may produce early stop codon or frameshift, leading to translation of dysfunctional PELO protein. Therefore, in the first batch of plant transformation, four gRNAs were selected to disrupt the genome region of the eRF1_1 domain in the *SlPelo* locus that would eventually abolish the function of PELO protein. (Figure 1b, Figures S1 and S2).

Recent studies of the *Mlo* gene family in tomato suggested that *SlMlo1* is the *susceptibility* gene involved in developing powdery mildew disease. Previously developed loss-of-function *SlMlo1* mutant plants developed by RNAi and CRISPR/Cas9 showed strong resistance against powdery mildew disease-causing fungus *Oidium neolycopersici* [24,27]. Therefore, two gRNAs for targeting the *SlMlo1* locus were chosen that predicted the formation of optimal secondary structures with limited or no off-target properties, among which Mlo1-gRNA 2 showed efficient editing in the previous report [24]. The selected target sites were located at exon 11 of the *SlMlo1* and were spaced 73 bp apart from each other, intending to generate large deletions intervening in the Mlo1-gRNA 1 and Mlo1-gRNA 2 (Figure 1c,d).

**Figure 2:**
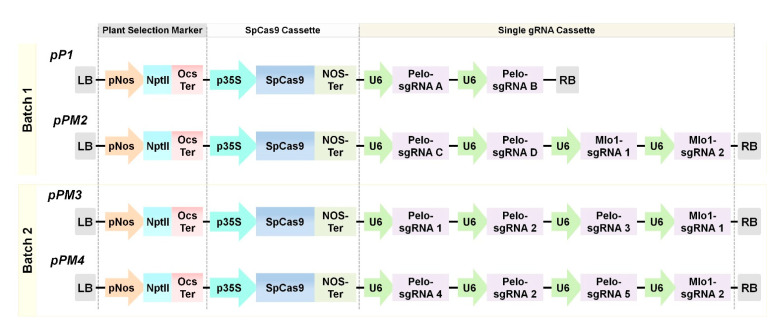
Schematic of the multiplex CRISPR/Cas9 binary transfer-DNA (T-DNA) vector cassettes (pP1, pPM2, pPM3, and pPM4). NPTII, kanamycin resistance gene under pNos promoter and tOcs terminator; p35S, promoter of 35S CaMV (cauliflower mosaic virus); pU6, promoter of AtU6; SpCas9, wild-type *Streptococcus pyogenes* Cas9; sgRNAs for *SlPelo* and *SlMlo1*; vector backbone pAGM4723.

The sgRNA expression cassettes, functional SpCas9 expression cassette, and plant selection marker (kanamycin) were combined into the binary transfer-DNA (T-DNA) vectors using Golden Gate cloning [28] (Figure 2). Ultimately, *Agrobacterium*-mediated tomato transformation was carried out to deliver CRISPR/Cas9 components for the generation of genome-edited BN-86 lines.

### 2.2. Generation of Genome-Edited Tomato Plants

The tomato cotyledons were subjected to *Agrobacterium*-mediated transformation. The tomato tissue culture, including the callus generation, shoot induction, and elongation, were performed using optimized culture media described in the Materials and Methods section. The elongated shoots grown on the kanamycin selection medium were transferred into a rooting medium to allow root development. The genomic DNA was extracted from the leaves of regenerated plants, i.e., genome-edited generation 0 (G0), and analyzed for the T-DNA presence by polymerase chain reaction (PCR) amplification using *SpCas9* gene-specific primers. Total 8 and 28 transformed events showed T-DNA integration in pP1 and pPM2, respectively (Figure S3). Although editing at *SlMlo1* target sites was observed in pPM2-derived plant events, editing in the *SlPelo* locus was not detected in the transformed events of pP1 and pPM2 constructs. In the second batch of transformation, another set of five gRNAs were designed for *SlPelo* editing according to specific criteria (Figure 1b,c). The secondary structures of all the gRNAs together with scaffold sequence (single gRNA, sgRNA) were predicted (Figure S2) using the Mfold program [29] to analyze interactions between gRNA and scaffold region. The multiple gRNAs for each targeted locus were combined in T-DNA vectors to trigger the deletion of larger fragments.

Among the analyzed 43 regenerated plants, 24 plants carried *SpCas9* from the batch of a co-cultivated mix of pPM3 and pPM4, which exhibited the transformation efficiency of 55.81% (Figure S4a). The regenerated G0 plants were analyzed for possible editing in *SlPelo* and *SlMlo1* by delivered CRISPR/Cas9 tool. Among the examined plants, *SpCas9*-positive 24 lines were chosen for further genotyping. PCR amplification of the target region of *SlPelo* and *SlMlo1* was resolved on the agarose gel. The amplicon products of similar size were observed from the analyzed wild-type (WT) and regenerated G0 plants (Figure S4b). Furthermore, Sanger sequencing of the direct PCR product was performed to reveal the mutation patterns in the independent G0 plants. Sanger sequencing results exhibited mixed reads from a single sample. Therefore, the Inference of CRISPR Edits (ICE) tool was used to decompose the Sanger sequencing data [30]. Out of 24 *SpCas9*-positive plants from pPM3+pPM4 transformation, 3 plants (G0-36, G0-37, and G0-41) showed mutation at the target site 1 in *SlPelo* (Figure S5a), whereas indel mutations were not detected in the *SlMlo1* region of all three events. On the other hand, some of the *SpCas9*-positive plants showed editing only in the *SlMlo1* locus. Therefore, the results of the characterization of the genome-edited lines for the *SlPelo* and *SlMlo1* locus are summarized in the independent sections for clarity.

### 2.3. Generation of SlPelo-Edited Tomato Plants

Analysis of transformed events indicated that one nucleotide base was inserted at the SpCas9 cutting position of Pelo-gRNA 1, i.e., 3 bp upstream of the PAM. The presence of mutations suggested that efficient somatic mutation occurred in the G0 generation. Therefore, three events (G0-36, G0-37, and G0-41) were characterized in the G1 generation. The self-crossed *SlPelo* G1 lines were generated, and then we investigated the editing pattern. For genotyping, the genomic DNA was extracted from the leaf samples, and the target region of *SlPelo* was PCR amplified (Figure S4d). Sanger sequencing and ICE analysis of screened G1 plants showed efficient editing in a single G0-41 line at the target site 1 in *SlPelo* locus. The biallelic mutations with 22 to 100% editing efficiency were observed (G1-41-39 (95%), G1-41-40 (99%), G1-41-41 (22%), G1-41-42 (99%), G1-41-43 (100%), G1-41-44 (100%), and G1-41-46 (47%)) in G1 plants (Figure 2a,b and Figure S5b). Overall, six plants were biallelic knockout, one was chimeric knockout, and one was heterozygous among the generated 11 plants from the G0-41 event, giving editing efficiency of 54.5%, 9.09%, and 9.09%, respectively, in G1 generation. Interestingly, high editing efficiency was found in G1 plants with additional mutation alleles. The obtained mutation patterns in G1 plants were different from the G0 stage, suggesting the active somatic mutations occurred at developmental stages. Further, analysis of G1 plants showed the presence of *SpCas9* gene in the G1 stage, indicative of continuous expression of CRISPR components in G0 and G1 generation facilitating different editing patterns.

### 2.4. TYLCV Resistance in CRISPR/Cas9-Edited SlPelo Plants

**Figure 3:**
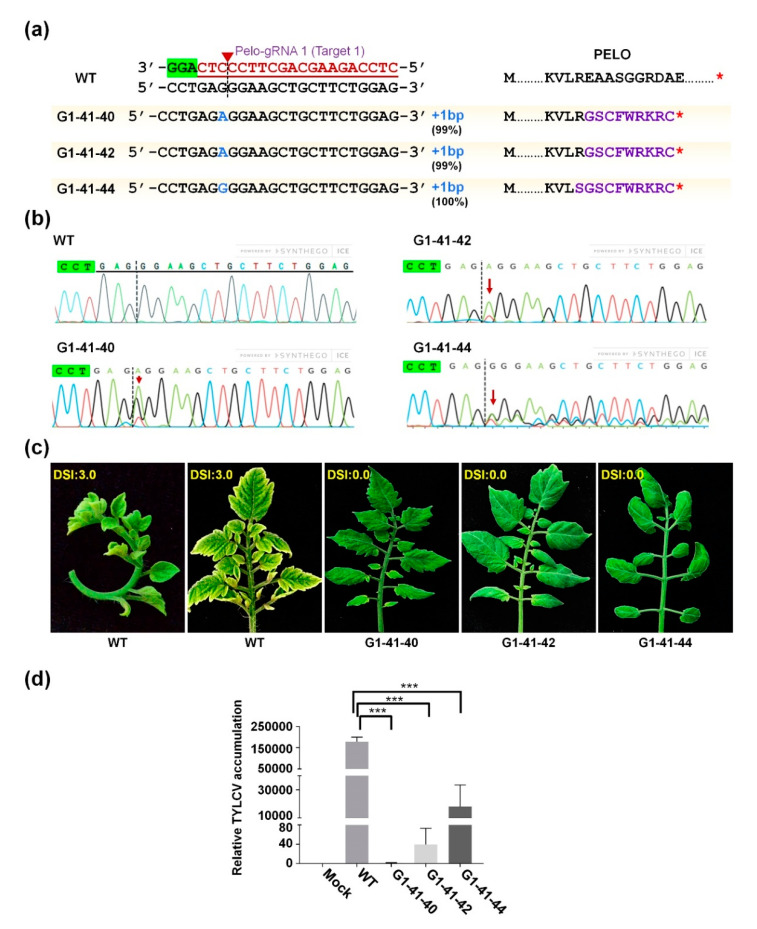
Generation of CRISPR/Cas9-mediated *SlPelo* genome-edited tomato lines resistant to *Tomato yellow leaf curl virus* (TYLCV). (**a**) Target site sequences in the wild-type (WT) and three *SlPelo*-knockout G1 plants (G1-41-40, G1-41-42, and G1-41-44) showing the mutation pattern with 1 bp insertion (blue) at the SpCas9 cleavage site. The knockout efficiency (%) of individual lines was evaluated using the Inference of CRISPR Edits (ICE) tool. The right panel shows WT and truncated PELO amino acid sequence, separately. The altered amino acid sequence is depicted in purple and stop codon is depicted as a red star; (**b**) Chromatograms showing the mutant *SlPelo* alleles (red arrow). The dotted line depicts the sgRNA cleavage position; (**c**) Resistance analysis of *SlPelo* mutant lines tested for TYLCV pathogenicity. The phenotype of the G1 tomato plants evaluated at 28 days post-infection (DPI) with TYLCV-virulent factor. According to visual TYLCV symptoms, the Disease Severity Index (DSI) was calculated from three replicates; (**d**) Relative DNA amounts of TYLCV in WT and mutant tomato plants germinated from leaves collected from TYLCV-inoculated tomatoes. Individual samples (three biological replicates) for each plant were used for analysis. Relative TYLCV DNA amounts were normalized against the tomato *SlEF1α* gene as an internal control. Data analyses were conducted using the 2^−ΔΔ*Ct*^ method. Error bars represent standard error (SE). Data represent three biological replicates. Asterisks indicate statistically significant differences (*** *p* < 0.0001, independent samples *t*-test).

**Figure 4:**
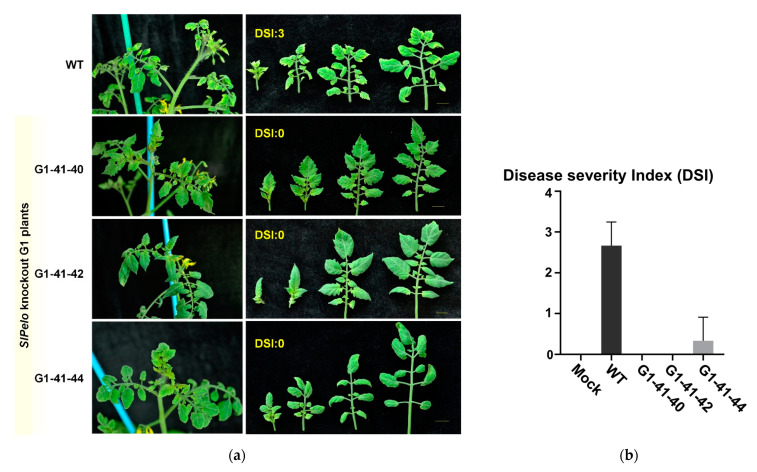
TYLCV-resistant phenotypes of CRISPR/Cas9-edited tomato plants. (**a**) TYLCV-inoculated wild-type (WT) and *SlPelo*-knockout plants were evaluated for TYLCV disease symptoms; (**b**) Disease Severity Index (DSI) was calculated for WT and *SlPelo*-knockout G1 plants (G1-41-40, G1-41-42, and G1-41-44). The photos were taken 28 days post-infection.

The genome-edited *SlPelo*-knockout mutants were tested for resistance against TYLCV. Three biallelic knockout mutant plants (G1-41-40, G1-41-42, and G1-41-44) were infected using *Agrobacterium*-carrying TYLCV virulent factor (pCAM-TYLCV-1.7mer). TYLCV infection symptoms started to appear after three weeks of post-infection in WT-like plants. TYLCV symptoms were expanded at 28 days post-infection (DPI), and all the newly emerged WT leaves showed yellowing and curling leaf margins. The tested G1-41-40 and G1-41-42 plants exhibited no visible TYLCV symptoms (Figure 3c and Figure 4). In contrast, slight curling of leaves from margins but not the yellowing symptoms were observed in the G1-41-44 plant. The Disease Severity Index (DSI) values were calculated according to the severity of visual TYLCV symptoms (Figure 4a). TYLCV disease symptoms vary depending on the plant growth stage, environmental conditions, [31], and viral load [13]. Similarly, different TYLCV symptoms, such as severe yellow leaf margins, curling, or both (yellow leaf margins with curling), were observed in infected WT plants (Figure 4b). Mock control, inoculated WT, G1-41-40 line, G1-41-42, and G1-41-44 showed DSI values of 0.0, 2.5, 0,0, 0.0, and 0.0, respectively.

The copy number of TYLCV was evaluated by real-time quantitative PCR (qPCR) in four-week-old leaves to know the TYLCV multiplication and spread to other plant parts. The designed qPCR primers can specifically detect viral components (pCAM-TYLCV-1.7mer) [32]. Thus, amplified product amount correlates with viral DNA copy number. TYLCV was detected in inoculated WT and *SlPelo*-mutant plants except for the mock control sample. The qPCR data demonstrated that viral titer in all the *SlPelo*-mutant plants was significantly low compared with susceptible inoculated WT plants (Figure 3d). G1-41-40 and G1-41-42 knockout mutant lines showed negligible viral titer; however, the G1-41-44 line revealed a detectable number of viral traces. The viral load and DSI score (symptoms) were found to be correlated and suggested that low TYLCV titer restricted TYLCV replication in *SlPelo*-knockout plants.

Even though off-targeting in plants is not a significant concern [33], potential off-target sites were also analyzed in the G1-41-40 line, despite the G1 plants not exhibiting any growth penalty. The genomic regions of potential off-target sites predicted by the Cas-OFFinder program [34] were PCR-amplified with target-specific primer pairs (Table S1). According to the program, Pelo-gRNA 1 did not show any off-target site in the tomato genome. Generally, an off-target site with two mismatches in the seed region of protospacer (for example, off-target-2-1) had less chance to be recognized by on-target sgRNA-Cas9 complexes [19]. Altogether, Sanger sequencing analysis data showed no undesired mutations in tested off-target sites (Table S2).

### 2.5. Characterization of SlMlo1-Knockout Mutants for Powdery Mildew Resistance

**Figure 5:**
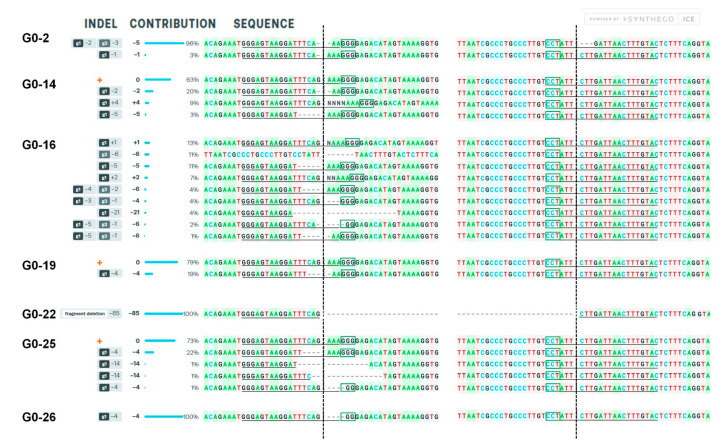
PCR-amplified *SlMlo1*-target region of G0 lines was Sanger sequenced and decomposed using the ICE tool to determine the type of mutation (indel) and editing efficiency (contribution) in the analyzed population. Dotted vertical lines denote target cleavage sites. Dashes imply nucleotide deletions.

Tissue culture-produced plants (G0) were analyzed for stable *SpCas9* presence using gene-specific primer pair (Figure S4a,c). The *SpCas9*-positive tomato lines were further examined for genotyping. The electrophoretic separation of PCR-amplified *SlMlo1* target region demonstrated the same size amplicon for 10 G0 plants except for one line (G0-22) that showed a smaller size amplicon than WT (Figure S4c). Further, Sanger sequencing of PCR products was analyzed with SnapGene and the ICE tool (Figure 5).

Among the analyzed plants, editing at all the target sites (G0-2, G0-14, G0-16, G0-19, G0-22, and G-26) were observed, except G-25, which generated mutation in only Mlo1-gRNA 1 target site. Biallelic or multiple mutation patterns were dominantly obtained in the G0 plants. Notably, the G0-22 line showed efficient editing at both target sgRNA site that produces 85 bp deletion from the cleavage site after SpCas9 sgRNA cuts 3 bp upstream from the PAM. G0-22 line showed homozygous mutation pattern in the G0 stage and hence was taken to the next generation. 

**Figure 6:**
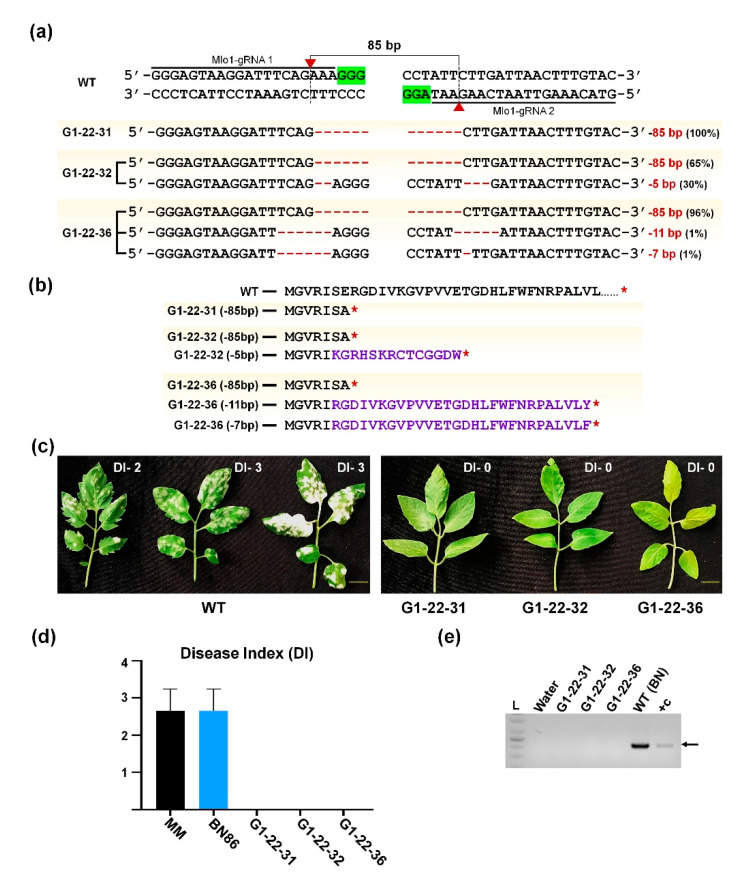
Characterization of CRISPR/Cas9-mediated *SlMlo1* genome-edited tomato lines for powdery mildew resistance. (**a**) Indel patterns of three *SlMlo1*-knockout plants showing the homozygous (G1-22-31), biallelic (G1-22-32), and chimeric (G1-22-36) genotype. The knockout efficiency (%) of individual lines evaluated using the ICE tool. Red dash indicates the deleted nucleotides; (**b**) Comparison of amino acid sequence between wild-type (WT) MLO1 protein and truncated region resulting from knockout alleles. Stop codon in red star symbol and altered amino acids in blue was indicated; (**c**) Analysis of *SlMlo1*-knockout mutant lines tested for resistance against powdery mildew-causing fungus *Oidium neolycopersici*. The phenotype of the mutant plants evaluated at 21 days post-infection (DPI). Referring to visual fungal growth symptoms, we calculated the disease index; (**d**) Powdery mildew disease index was calculated with WT (BN-86 and Moneymaker (MM)) and G1 *SlMlo1* mutant lines (G1-22-31, G1-22-32, G1-22-36). Error bars represent SE (three biological replicates); (**e**) Detection of *O. neolycopersici* by PCR method using strain-specific 16S ribosomal RNA (rRNA) primers. Non-infected plants used as mock control; fungal DNA used for PCR as a positive control.

*SlMlo1* mutant G0-22 line was self-crossed to generate G1 plants that were further characterized. Nine G1 plants were genotyped for mutation analysis and stable *SpCas9* gene presence (Figure S6). The amplified PCR products of the *SlMlo1* target region were analyzed by the agarose gel electrophoresis and further sequenced using Sanger sequencing. One homozygous, one biallelic, five chimeric, and two heterozygous plants were obtained in G1 generation with editing efficiency 11.11%, 11.12%, 55.55%, and 22.22%, respectively. Among the examined G1 lines, one knockout line (G1-22-31) was identified as a homozygous carrying the mutant allele of 85 bp deletion. The other two knockout lines (biallelic G1-22-32 and chimeric G1-22-36) had an 85 bp deletion with an additional deletion allele (Figure 6a and Figure S7). These nucleotide deletions resulted in a frameshift of the open reading frame (ORF) that created premature translation termination in the MLO1 polypeptide in knockout mutant plants (Figure 6b).

### 2.6. Powdery Mildew Resistance Test in CRISPR/Cas9-Edited SlMlo1 Plants

Tomato transmembrane protein MLO1 is involved in the interaction between tomato and powdery mildew fungal pathogen. Three independent *SlMlo1*-knockout lines, namely, homozygous (G1-22-31), biallelic (G1-22-32), and chimeric (G1-22-36) plants, along with WT-like control plants, were challenged for powdery mildew disease-resistance assay (Figure 6c). The fungal inoculum was sprayed in tested plants, and the fungal disease occurrence was monitored. All three *SlMlo1* mutant plants showed complete resistance against powdery mildew causing pathogen *Oidium neolycopersici*, whereas WT-like plants showed disease symptoms that scored in a disease index assay (Figure 6d).

Furthermore, PCR analysis of non-inoculated and inoculated plant leaves was checked for fungal pathogen presence using 16S rRNA-specific primers (Figure 6e; Table S1). Pathogen traces were detected in WT-inoculated samples, whereas they were absent in tested knockout plants. Additionally, potential off-target mutations generated by CRISPR/Cas9 were also evaluated in the G1-22-32 line. The selected off-target regions predicted by the Cas-OFFinder tool were PCR amplified, Sanger sequenced, and aligned with native sequence (Table S2). The four off-target sites (off-target-6-1, off-target-6-2, off-target-6-3, and off-target-7-1) had two or more mismatches in the seed region of gRNA, and therefore they may not cleave the DNA due to unstable interactions with the sgRNA–Cas9 complex [19]. Other off-target sites (off-target-7-2 and off-target-7-3) also showed no off-target effect in the tested line (Table S2).

## 3. Discussion

The targeting of *S*-genes in crop species is a promising approach as opposed to transgenics in order to circumvent the regulatory hurdles and environmental concerns owing to stable overexpression of *R*-genes or CRISPR components [35]. A variety of CRISPR-based tools enable the precise editing of desirable loci in the plant genome [36]. In this study, we targeted two distinct *S*-genes (*SlPelo* and *SlMlo1*) using the CRISPR/Cas9 tool in the tomato BN-86 line. Initially, we designed plasmid vectors carrying sgRNAs for simultaneous targeting of both the genes to generate single or double mutant plants to avoid the tedious plant transformation process. The use of multiple gRNAs to edit the single gene increases the chance of large deletions and knockout generation [37]. In case of *SlMlo1*, co-expression of two sgRNAs generated larger deletion. However, synchronized editing at both the gRNAs in *SlPelo* gene was not observed in the earlier screening of several events. Some previous reports suggested that the simultaneous targeting of multiple loci might decrease the genome editing efficiency due to various reasons [38]. For instance, lower sgRNA activities targeting the *SlPelo* gene compared to *SlMlo1* gRNAs. Out of nine gRNAs (Figure 1 and Figure S1), only one gRNA (Pelo-gRNA 1) showed successful editing at target site 1 in the *SlPelo* locus.

The lower efficiency of gRNAs can be attributed to several reasons, for example, highly unstable Cas9–gRNA complexes and stable occupancy of inactive sgRNA with Cas9 [39]. Thus, accidentally chosen inactive sgRNA may reduce the activity of the active-sgRNA and may decrease overall genome editing efficiency. Secondly, chromatin confirmation may significantly affect the accessibility of target sites and not be considered in sgRNA designing tools [40]. Thirdly, a recent report indicated Cas9 availability as one of the limiting factors in multiplexing studies [41]. Several sgRNAs were expressed simultaneously in the present work, and thus it is possible that gRNA activities were compromised due to increased competition for SpCas9 binding. Although gRNAs were chosen on the basis of specific criteria from predicted secondary structures, the algorithms of plant gRNA prediction tools are based on data generated from gRNA datasets of non-plant hosts [40]. Moreover, predicted outcomes do not necessarily reflect the actual in vivo sgRNA activity. Single or combination of one of the above-discussed factors might be contributing to lower editing at *SlPelo* locus.

The TYLCV disease is one of the most devastating viral diseases in tomatoes. Attempts to control the disease with conventional methods, such as breeding and transgenic approaches, have met with limited success [5]. Here, we demonstrate that the CRISPR/Cas9-mediated commercial tomato BN-86 line targeting *SlPelo* to generate TYLCV-resistant tomato plants. We characterized three independent *SlPelo*-knockout G1 plants that exhibited 1 bp insertion at the DSB site of SpCas9, i.e., 3 bp upstream of the PAM site [19,42]. All the ORF-coding region mutations created early stop codons, causing the formation of truncated PELO proteins and ultimately generated the loss-of-function alleles for the *SlPelo* gene (Figure 3). The *SlPelo* mutant G0 plants were biallelic or mosaic, with low indel frequencies (>10%) in coding exon. Nevertheless, screening of segregating G1 generation plants successfully produced biallelic knockout mutants. Among the G0 plants, only one line (G0-41) was found to carry the mutant allele in the G1 generation, and others showed WT allele (Figure S4d). Such a phenomenon may be possible due to lower editing efficiency in somatic cells of G0 generation that could not be carried by all the reproductive tissues generating seeds for the next generation. Improved editing efficiency with additional mutant alleles found in G1 plants suggested that the G0 plants-carrying T-DNAs continually expressed the SpCas9–sgRNA complexes throughout the developmental stages [42]. Depending on CRISPR/Cas9 editing efficiency on homologous chromosomes, editing outcomes can be monoallelic (single), biallelic (double), or chimeric (more than two). Inherited biallelic mutation pattern has been reported in the CRISPR experiments in plants, for instance, rice [38], tomato [42], Arabidopsis [43], and *Medicago truncatula* [44]. Thus, our knockout mutant plants can be readily inherited to the next generation without affecting the normal physiological traits.

Previous studies suggested that *SlPelo* is a crucial factor in TYLCV-mediated disease development. RNAi-mediated silencing of *SlPelo* interrupted viral proliferation and generated TYLCV-resistant phenotype [8]. We challenged our *SlPelo*-edited knockout G1 plants against TYLCV. Consistent with the previous report, we detected typical TYLCV symptoms and TYLCV DNA copy accumulation in inoculated WT-like plants. However, G1 plants (G1-41-40, G1-41-42) carrying mutations in *SlPelo* were TYLCV-symptomless. Significant reduction in TYLCV DNA load was observed in all the tested mutant plants compared with an inoculated WT-like plants.

Moreover, mild symptoms in G1-41-44 may be caused due to higher inoculation load or partial loss-of-function of PELO. Our study used virulent factor for disease assay that mimicked the natural whitefly-mediated transmission of TYLCV. In natural conditions, many factors can influence the TYLCV pathogenicity, such as virus genome, systemic movement of viral components, and viruliferous (virus-carrying) whitefly population [45]. *Agrobacterium*-mediated transmission of high viral particle doses also failed to produce TYLCV resistance barrier in *SlPelo*-knockout mutant lines, suggesting that the strong interaction with PELO and TYLCV was elucidated. Our study may open up new research possibilities that can reveal possible interactions between plants and viral disease development. Infection of TYLCV is orchestrated by interaction between three factors, namely, whitefly, plant, and TYLCV. Therefore, complementing genome-edited lines with the whitefly control through minimal use of pesticides may help to combat the spread of TYLCV disease. This approach is reliable and durable to develop TYLCV resistance. The elite tomato BN-86 line used in the study does not carry the *Ty-5* resistant allele as reported for some TYLCV-resistant cultivars [8,46]. Another way to improve TYLCV resistance in tomato cultivars is by pyramiding TYLCV resistance genes in breeding programs using genetic markers [46].

To integrate the powdery mildew resistant trait in the Korean commercial BN-86 cultivar, we targeted the *SlMlo1* using the SpCas9-mediated CRISPR/Cas9 system. Previous reports suggested that loss-of-function mutation of *SlMlo1* produces complete disease resistance against powdery mildew [24,27]. To evaluate disease pathogenicity, we tested our *SlMlo1*-knockout mutant plants against powdery mildew pathogen *O. neolycopersici*. Our *SlMlo1* mutant lines (G1-22-31, G1-22-32, and G1-22-36) demonstrated complete resistance against powdery mildew, whereas inoculated WT-like plants showed severe disease symptoms as previously reported in Moneymaker cultivar [27]. The resistant phenotype in *SlMlo1* mutant lines indicates that the *SlMlo1* function is conserved irrespective of the genetic background of the BN-86 line. We obtained double targeted mutant plants (*SlPelo/ SlPelo SlMlo1/+*) in the recent screening of additional events (data not shown) and continued our work to develop the knockout double mutant BN-86 lines. We also found that many of our G1 mutant plants still possessed the transgene (*SpCas9*), suggesting multiple T-DNA integration in G0 plants. The segregating population can produce transgene-free genome-edited plants. Furthermore, T-DNA-free plants will be subjected to off-target analysis to avoid the undesirable mutations in other genetic locations.

## 4. Materials and Methods

### 4.1. Characterization of SlMlo1-Knockout Mutants for Powdery Mildew Resistance

The seeds of *Solanum lycopersicum* BN-86 line were received from Bunongseed Co., Ltd. (Gimje, Korea). The seeds were surface sterilized in 70% ethanol for 1-2 min and then placed in a gentle vortex with Clorox solution, 30% sodium hypochlorite (NaOCl, Daejung Chemical and Metals Co. Ltd., Busan, Korea), and 1 drop of Triton X-100 (Amresco, Solon, OH, USA) for 20 min. Sterilized seeds were rinsed 5 times with double-distilled autoclaved water. The seeds were placed for germination on a half-strength Murashige and Skoog (MS) medium containing 20 g/L sucrose and 15 g/L plant agar. Initially, the seeds were kept in the dark for 4 days and then moved to light for 3-4 days. The culture condition was 25 ± 2 °C with 16-h light and 8-h dark cycle under cool fluorescent light.

### 4.2. Plant sgRNA Designing, Secondary Structure, and Off-Target Prediction

The full-length genomic sequences of the *SlPelo* and *SlMlo1* loci were retrieved from the Sol Genomics Network database [47]. The PELO protein sequence from the Plaza database (https://bioinformatics.psb.ugent.be/plaza/ accessed on 10 February 2021) was used to predict protein motifs using the NCBI-CDD tool (www.genome.jp/tools/motif/NCBI-CDD accessed on 10 February 2021). The gRNAs were selected (Table S1) using CRISPR-P 2.0 tool [48] with defined criteria (small nucleolar RNA (snoRNA) promoter: U6; Guide Sequence Length: 20, Target Genome: Solanum lycopersicum SL3.0). The secondary structure of the sgRNAs was predicted using the Mfold web server with a temperature of 28 °C [29]. The potential off-target sites of each sgRNA were analyzed by the Cas-OFFinder tool [24], and the gRNAs with no or fewer potential off-target sites were preferred.

### 4.3. Plasmid Construction

All the constructs were cloned and assembled using the Golden Gate assembly method in *Escherichia coli* 10-beta cells [28,49]. Desired level 0 and 1 module were PCR-amplified using specific primer pair (Table S2) and high-fidelity DNA polymerase (Phusion Taq, Thermo Fisher Scientific, Waltham, MA, USA). PCR conditions were as follows: 98 °C for 30 s, followed by 32 cycles of denaturing at 98 °C for 30 s, annealing from 62 °C for 30 s, 72 °C extensions for 30 s, and then final extension at 72 °C for 5 min. All PCR products were cleaned using HiGeneTM Gel and PCR purification kit (BioFact Co. Ltd., Daejeon, Korea). The sgRNA expression was driven by Arabidopsis U6 (AtU6) promoter (pICSL01009, Addgene #46968) and terminated by T-repeats (7 Ts). The level 1 expression units, including plant selection marker (kanamycin), humanized *SpCas9* (Addgene #49771) expressed under constitutive promoter CaMV 35S, and sgRNA expression cassettes were assembled into the binary vector pAGM4723.

### 4.4. Plant Transformation

All the cloned binary vectors (Figure 2) were transformed into *Agrobacterium tumefaciens* GV3101 using electroporation. *Agrobacterium*-mediated transformation was used to deliver the CRISPR/Cas9 sgRNA components into the tomato. Explants for transformation were prepared from 7–8-day-old cotyledons (before the first true leaves). The cotyledons were sliced into small pieces (0.2–0.3 cm) from seedlings and used as explants. The explants were pretreated in the preculture medium (Murashige and Skoog (MS) basal salts, Gamborg B5 vitamins, 2.0 mg/L zeatin-riboside-trans isomer (ZR), 0.2 mg/L indolyl-3-acetic acid (IAA), 0.5 g/L 2-(*N*-morpholino)ethanesulfonic acid (MES), and 30 g/L glucose, pH 5.7) for 2 days. The precultured explants were pricked (8–10 prick/explant) and transformed using *Agrobacterium*-carrying CRISPR/Cas9 plasmids. Preculture of Agrobacteria was grown overnight in Luria–Bertani (LB) medium containing suitable antibiotics at 28 °C. Five percent of primary culture was inoculated into freshly prepared LB medium with suitable antibiotics for 4 h. An equal amount of culture from grown Agrobacteria carrying different vectors was mixed for co-transformation. Harvested cells were resuspended in MSB5 (MS basal salts, added with Gamborg B5 vitamins, pH 5.8) medium together with 150 µm acetosyringone and were used for tomato transformation. 

The co-cultivated explants were kept in the dark conditions at 25 °C for 2 days and then washed with timentin (300 µM) before transferring to shoot induction medium 1 (MS basal salts, Gamborg B5 vitamins, 2.0 mg/L ZR, 0.2 mg/L IAA, 0.5 g/L MES, and 30 g/L glucose, pH 5.7) containing kanamycin (60 mg/L) for 14 days. To achieve efficient regeneration, we carried out the subculture every 14-day interval in shoot elongation medium 2 (MS basal salts, Gamborg B5 vitamins, 1.0 mg/L ZR, 0.1 mg/L IAA, 0.5 g/L MES, 30 g/L glucose, and 60mg/L kanamycin, pH 5.7). The regenerated shoots were transferred to root induction medium (RIM) (MS basal salts, Gamborg B5 vitamins, 30 g/L glucose, 1.0 mg/L indole-3-butyric acid, 30 mg/L kanamycin, pH 5.7). Rooted plants were transferred to vermiculite pots for hardening before shifting to the greenhouse.

### 4.5. Plant Genomic DNA Extraction

The DNA was extracted from 100 mg leaf tissue using the cetyl trimethylammonium bromide (CTAB) method [50]. Briefly, leaf tissue was frozen in liquid nitrogen, and CTAB buffer was added to the crushed powder. After incubation for 1 h at 60 °C, the suspension was centrifuged, and the supernatant was transferred to a new Eppendorf tube. RNase solution A was added, and the mixture was incubated at 37 °C for 30 min. After that, genomic DNA was extracted using a protocol described in an earlier study [51].

### 4.6. Genotyping of Plants Progeny by PCR and Sanger Sequencing

The plant progenies were genotyped for potential editing by Sanger sequencing of PCR-amplified target region of *SlPelo* and *SlMlo1* locus. To identify the T-DNA presence in regenerated G0 and G1 plants, we designed PCR primers for amplification of partial DNA sequence of the *SpCas9* gene. *GAPDH* gene-specific primers were used as a positive control to validate the genomic DNA quality. The list of the primers, along with product length, is provided in Table S2. Approximately 50 ng of genomic DNA was used for PCR reaction of 25 µL. All the PCR conditions were applied according to manufacturer protocol (Phusion Taq, Thermo Fisher Scientific). The PCR amplicon was analyzed by Sanger sequencing using the services from Solgent Ltd. (Seoul, Korea) or Cosmogentech Ltd. (Seoul, Korea).

### 4.7. Analysis of Editing Efficiency by In Silico Method: SnapGene and Inference of CRISPR Edits (ICE) and TA Cloning

Sanger sequencing data were analyzed using SnapGene (GSL Biotech LLC, Chicago, IL, USA) and ICE (Inference of CRISPR Edits) tool. ICE (https://ice.synthego.com accessed on 10 February 2021) can decompose the Sanger sequencing data and generate the genome editing pattern in each sample [30]. The PCR products were cloned using a CloneJET PCR Cloning Kit (Thermo Scientific, Waltham, MA, USA) and analyzed by Sanger sequencing to identify individual editing patterns.

### 4.8. TYLCV Infection and Disease Indexing Assay

The self-crossed G1 homozygous *SlPelo* mutants and WT-like plants were grown at the greenhouse, and subsequently, axillary branches were used for micropropagation [52]. The micropropagated shoots were directly transferred to soil rite and covered with a plastic bag to maintain the humidity in greenhouse conditions. After 5-7 days, plants were subjected to TYLCV infection using an overnight grown culture of *Agrobacterium*-carrying virulent plasmid (pCAM-TYLCV-1.7mer). One drop of *Agrobacterium* culture was added to the apical shoot and gently pricked the area 5–10 times (repeated 2 times) using a needle and infiltrated into leaves by a needle-free syringe [32]. For mock control, LB with antibiotic was injected by a similar method. The TYLCV symptoms were evaluated using a Disease Severity Index (DSI) of 0 to 4, as described in the previous report [53].

### 4.9. Real-Time Quantitative PCR for TYLCV Titer Estimation

To estimate the copy number of TYLCV, we performed real-time quantitative PCR (qPCR) from the leaves of 28-day-old TYLCV-infected and mock control plants. The qPCR reactions were performed using the KAPA SYBR FAST qPCR kit (Kapa Biosystems, MA, USA) with specific primer sets (Table S1). Cycling of PCR consisted of pre-denaturation at 95 °C for 5 min followed by 40 cycles of a denaturation step at 95 °C for 10 min, an annealing step at 60 °C for 15 s, and an extension step at 72 °C for 20 s using the CFX384 Real-Time System (Bio-Rad, Hercules, CA, USA). The qPCR reactions with independent biological and technical replicates were performed thrice. Relative DNA amounts of TYLCV were normalized against *SlEF1 α*. Data analyses performed by the 2^−ΔΔ*Ct*^ method [54].

### 4.10. Oidium neolycopersici Inoculation, Powdery Mildew Disease Indexing Assay

The homozygous genome-edited plants and WT plants were inoculated with the fungal isolate: Pusan National University (PNU) of *O. neolycopersici* Strain PNU-003 (GeneBank: MW082786.1). The fungal strain was sprayed on 3-4-week-old plants with a suspension of conidiospores collected from previously infected tomato leaves of Moneymaker plants. White mycelial mats (hyphae, conidiophores, and considered) were brushed off from the adaxial sides of the infected leaves. A small number of brushed samples were mounted on a microscope slide with a drop of double-distilled water and examined using light microscopy. The spore suspensions used in the bioassays were prepared by diluting the specimens and were adjusted to 2.6 × 10^4^ spores/mL. These suspensions were spread 3 times on the whole plant at the 4–5 true-leaf stage. Inoculated plants were grown at 26  ±  2 °C with 70  ±  15% relative humidity and day length of 16 h in a greenhouse of PNU. To quantify the relative fungal biomass, we collected mock (control), WT-like, and genome-edited plant leaves at 21 days post-infection (DPI). After the formation of fungal growth on the WT plant’s surface, disease assays were conducted in accordance with the work of Bai et al. [27]. The intensity of fungal resistance was assessed on the basis of the Disease Symptom Index (DSI) as follows: DSI = 0, no symptoms; DSI = 1, symptoms covering less than 10% of the leaf area; DSI = 2, symptoms on 10 to 30% of the leaf area; and DSI = 3, symptoms covering more than 30% of the leaf area. Further, we performed the PCR reaction using *O. neolycopersici* fungal-specific 16S rRNA primers (Table S1) to evaluate fungal presence.

### 4.11. Off-target Assessment

The Cas-OFFinder tool (http://www.rgenome.net/cas-offinder/ accessed on 10 February 2021) was used to check for potential off-targets of the sgRNAs of *SlPelo* and *SlMlo1* [34]. The mismatch number was placed at 3 or less. The predicted off-target sites were analyzed for off-target mutations by PCR amplification and Sanger sequencing (Table S2).

### 4.12. Statistical Analyses

The results presented in the figures represent the average of three independent experiments. An independent-samples *t*-test evaluated the significance of the differences between the two datasets.

## 5. Conclusions

In conclusion, we used the CRISPR/Cas9 system to generate knockout mutants of the *SlPelo* and *SlMlo1* gene by *Agrobacterium*-mediated tomato transformation in elite tomato BN-86 line. The regenerated knockout *SlPelo* plants showed resistance to TYLCV infection. The loss-of-function of *SlPelo* restricted the TYLCV growth and did not produce any TYLCV symptoms in tested *SlPelo* mutant lines. Moreover, we successfully generated the *SlMlo1*-knockout mutant lines that demonstrated complete resistance against fungal powdery mildew disease. The development of double mutant (*SlPelo*/*SlMlo1*) transgene-free plants will be a step forward in multiplex genome editing-mediated introgression of biotic resistance traits in tomato. Furthermore, screening of segregating G2 and G3 generations of single and double mutants will produce the T-DNA-free lines to avoid the regulatory hurdles of GMOs. Results reported in the present study demonstrate the editing of *S*-genes as an effective approach to engineer the multiple pathogen resistance in tomato crop.

## 6. Patents

The patent application is being planned on the basis of the results from the work reported in this manuscript.

## Data Availability

All datasets supporting the conclusions of this article are included in the article and supplementary files.

## Supplementary Materials

The following are available online at https://www.mdpi.com/1422-0067/22/4/1878/s1: Figure S1. PELO amino acid sequence and conserved domain structure. Figure S2. Secondary structures of gRNA scaffold predicted using the Mfold tool. Figure S3. Genotyping of CRISPR/Cas9-regenerated G0 plants (Batch 1). Figure S4. Genotyping of CRISPR/Cas9-regenerated G0 and G1 plants (Batch 2). Figure S5. Sanger decomposition data of G0 and G1 *SlPelo*-edited lines. Figure S6. Screening of G1 tomato plants for editing of targeted loci in the tomato genome. Figure S7. CRISPR/Cas9-generated *SlMlo1*-edited alleles in the G1 generation. Table S1. List of the primers used in this study. Table S2. Guide RNA sequences used in present work and evaluated potential off-target sites.
