# Supplementary material for: CRISPR/Cas9-Mediated Generation of Pathogen-Resistant Tomato against Tomato Yellow Leaf Curl Virus and Powdery Mildew
Source: Int J Mol Sci. 2021 Feb 13;22(4):1878. doi: 10.3390/ijms22041878 (PMC7917697; doi:10.3390/ijms22041878)
Supplement: Supplementary file 1 [file ijms-22-01878-s001.pdf]

Supplementary Information

CRISPR/Cas9-mediated Generation of Pathogen-resistance Tomato against *Tomato Yellow Leaf Curl Virus* and Powdery Mildew

Dibyajyoti Pramanik [1,†], Rahul Mahadev Shelake [1,†,*], Jiyeon Park [2], Mi Jung Kim [1], Indeok Hwang [3], Younghoon Park [2,*], Jae-Yean Kim [1,*]

Affiliations

[1] Division of Applied Life Science (BK21 FOUR Program), Plant Molecular Biology and Biotechnology Research Center, Gyeongsang National University, Jinju 660-701, Korea; dpinbiotech@gmail.com (D.P.); ecastle109@gmail.com (M.J.K.)

[2] Department of Horticultural Bioscience, Pusan National University, Miryang 50463, Korea; o_omad@naver.com (J.P); ypark@pusan.ac.kr (Y.H.P.)

[3] R&D Center, Bunongseed Co., Ltd., Gimje 54324, Korea; usename@hanmail.net (I.H.)

[†] These authors contributed equally as co-first authors to this work.

***** Corresponding author: rahultnau@gmail.com (R.M.S.); ypark@pusan.ac.kr (Y.H.P.); kimjy@gnu.ac.kr (J.-Y. K.)

Phone: +82-55-772-1361 (J.-Y. K.)

Running title: CRISPR-mediated multi-pathogen resistance in tomato

Supplementary: Table of content

	Content	Page No
**Supplementary Figures**		
1.	PELO amino acid sequence and conserved domain structure.	1
2.	Secondary structures of gRNA-scaffold predicted using the Mfold tool	2
3.	Genotyping of CRISPR/Cas9-regenerated G0 plants (Batch 1)	3
4.	Genotyping of CRISPR/Cas9-regenerated G0 and G1 plants (Batch 2)	4
5.	Sanger decomposition data of G0 and G1 *SlPelo*-edited lines	5
6.	Screening of G1 tomato plants for editing at targeted loci in the tomato genome	6
7.	CRISPR/Cas9-generated *SlMlo1*-edited alleles in the G1 generation	
**Supplementary Table**		
1.	List of the primers used in this study	9
2.	RNA sequences used in present work and evaluated potential off-target sites	11

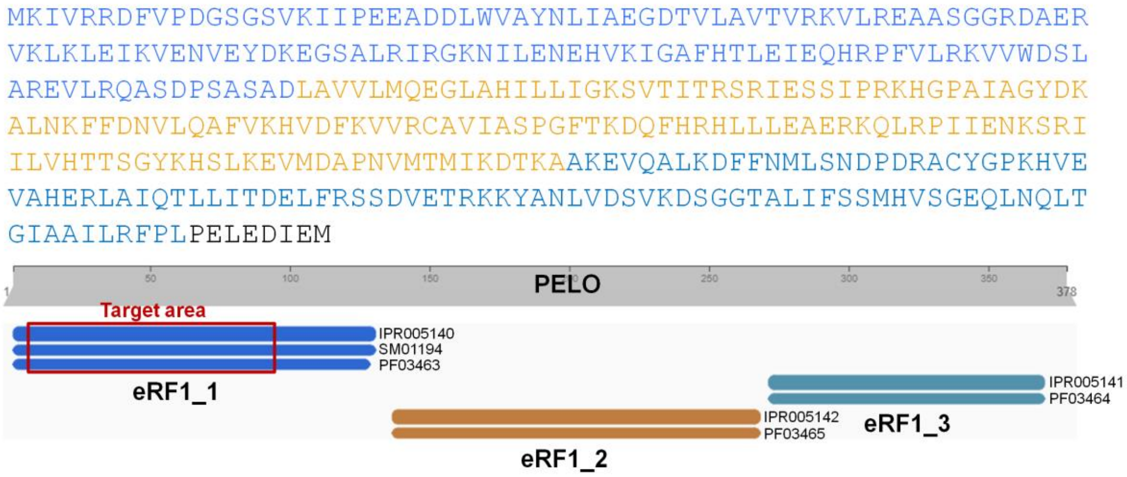

Figure S1.

PELO amino acid sequence and conserved domain structure.

PELO amino acid sequence and conserved domain structure. InterProScan scanning of the PELO polypeptide predicted to form three conserved eRF1 domains (eRF1_1, eRF1_2, and eRF1_3). The guide RNAs (gRNAs) were designed for targeting the eRF1_1, depicted with a red box.

(a)

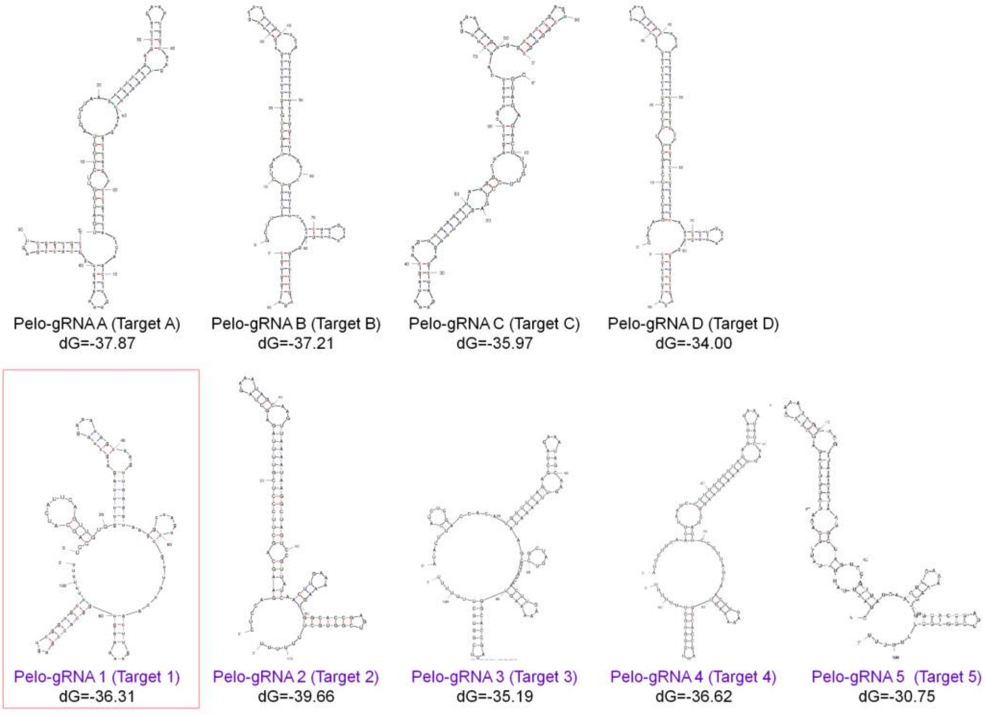

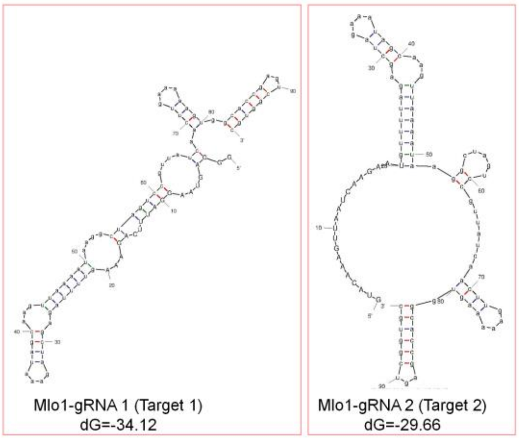

(b)

Gene	sgRNA	TSL	GSL	CBP	TBP	IBP	GC %	Source
SlPelo	Target A	3	0	6	11	0	40	This study
	Target B	3	0	5	10	0	45	
	Target C	3	0	6	11	0	35	
	Target D	3	0	3	11	0	40	
	Target 1	3	0	5	11	0	60	
	Target 2	3	0	3	5	0	40	
	Target 3	3	0	3	7	0	50	
	Target 4	3	0	4	4	0	25*	
	Target 5	3	0	2	3	0	20*	
SlMlo1	Target 1	3	0	5	7	0	40	
	Target 2	3	0	5	6	0	25*	Nekrasov et al., 2017

* GC <30%

Figure S2.

Secondary structures of gRNA-scaffold predicted using the Mfold tool.
(a) Target sgRNA secondary structure
(b) Parameters calculated from the predicted secondary structures of sgRNAs. TSL: total stem loop; GSL: Stem loop in the guide sequence; CBP: consecutive base pair (guide sequence and the other sequence); TBP: total base pairs in guide sequence with other sequence; IBP: internal base pairs in gRNA; GC content (%) of gRNA. Further details about the criteria available on the webpage of the CRISPR-P 2.0 tool.

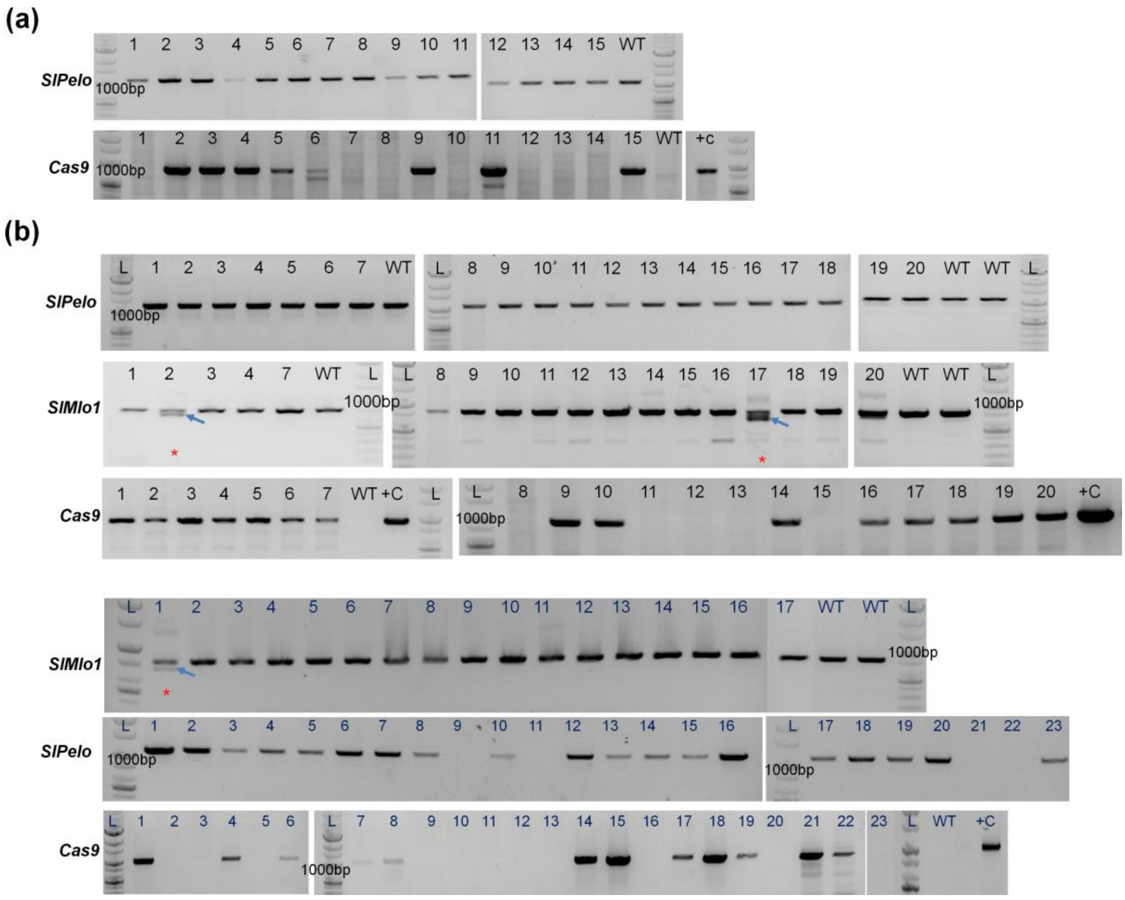

Figure S3.

Genotyping of CRISPR/Cas9-regenerated G0 plants (Batch 1).

Genotyping of G0 plants generated from (a) pP1 and (b) pPM2 for T-DNA integration (*SpCas9*) and target gene modifications. The *SpCas9*-positive independent G0 lines were analyzed in agarose gel electrophoresis after PCR amplification of the targeted genomic region. WT- targeted genomic region amplified from wild-type plant. Purified T-DNA plasmid was used as a positive control (+C) for *SpCas9* in PCR experiments. The plant line shows a shorter amplicon due to possible large deletion in the targeted region indicated using a blue arrow and red star symbol.

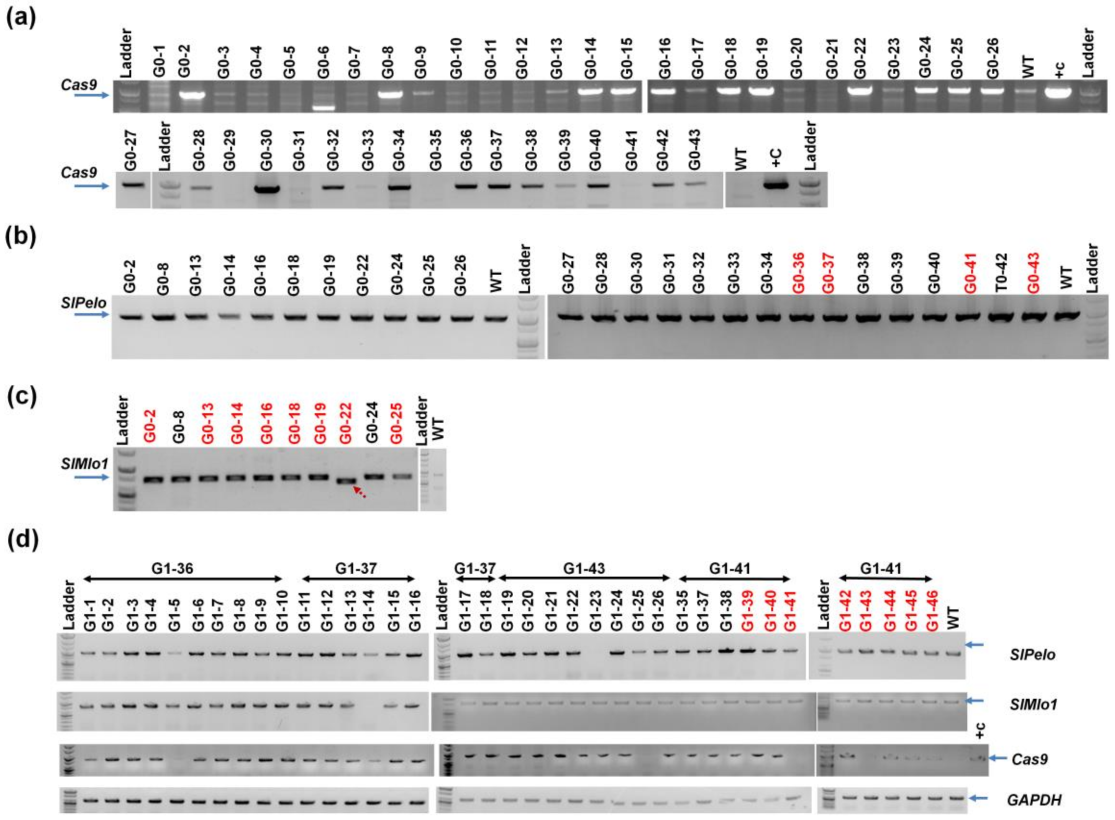

Figure S4.

Genotyping of CRISPR/Cas9-regenerated G0 and G1 plants (Batch 2).

(a) G0 plants genotyped for stable Cas9 integration alongside wild type and positive control (SpCas9-L2 plasmids);

(b, c) Agarose gel electrophoresis of PCR-amplified fragments of *SlPelo* and *SlMlo1* from the genome DNA of *SpCas9*-positive G0 lines, including wild-type (WT). The blue arrow indicating the size of the expected PCR product, the red dotted arrow indicates band shift due to large deletion.

(d) Screening of genome-edited plants in G1 generation. The G1 plants were genotyped for possible target gene modification and transgene presence. PCR-amplified target regions of *SlPelo* and *SlMlo1* locus separated on agarose gel electrophoresis. Purified T-DNA plasmid was used as a positive control (+C) for *SpCas9* in PCR. *GAPDH* used as an internal control gene.

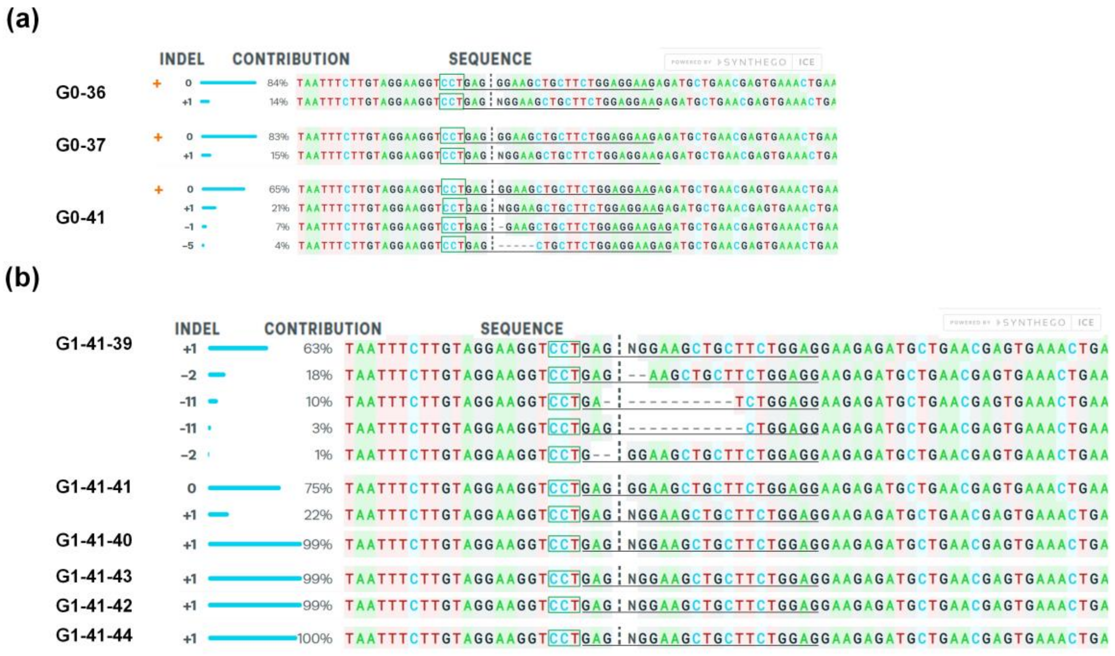

Figure S5.

Sanger decomposition data of G0 and G1 *SlPelo*-edited lines.
The gRNA target area of respective (a) G0 and (b) G1 plants was PCR amplified, sequenced with Sanger sequencing, and evaluated with the ICE tool. Indel and contribution showing the type of mutation and editing efficiency in the analyzed population, respectively. Dotted vertical lines denoting the target cleavage sites. Dash indicating deleted nucleotide bases. 'N' indicating the presence of mix chromatogram pick. Nucleotide deletions are implied by dashes.

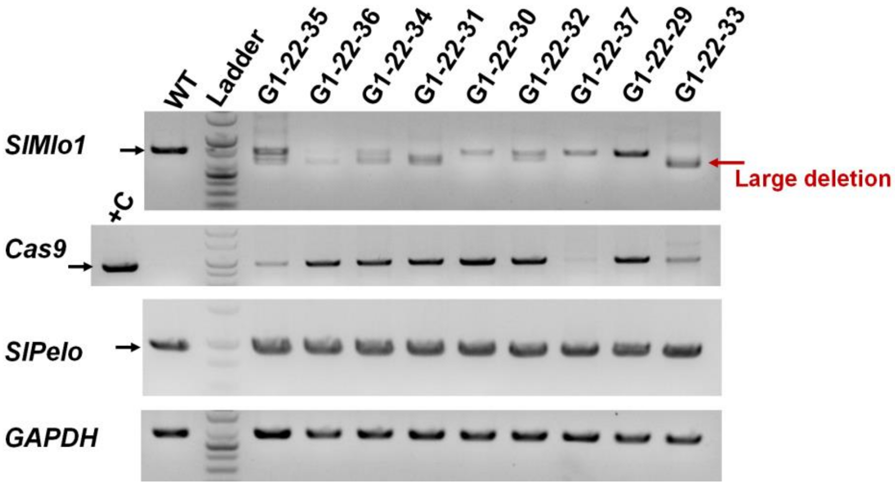

Figure S6.

Screening of G1 tomato plants for editing of targeted loci in the tomato genome.
Line number G0-22 was self-crossed and produced G1 plants genotyped for transgene presence (*SpCas9*) and possible editing of target genes. Agarose gel electrophoresis showing the PCR-amplified target region. Purified T-DNA plasmid used as s positive control (+C) for *SpCas9* in PCR experiments. *GAPDH* used as an internal control gene.

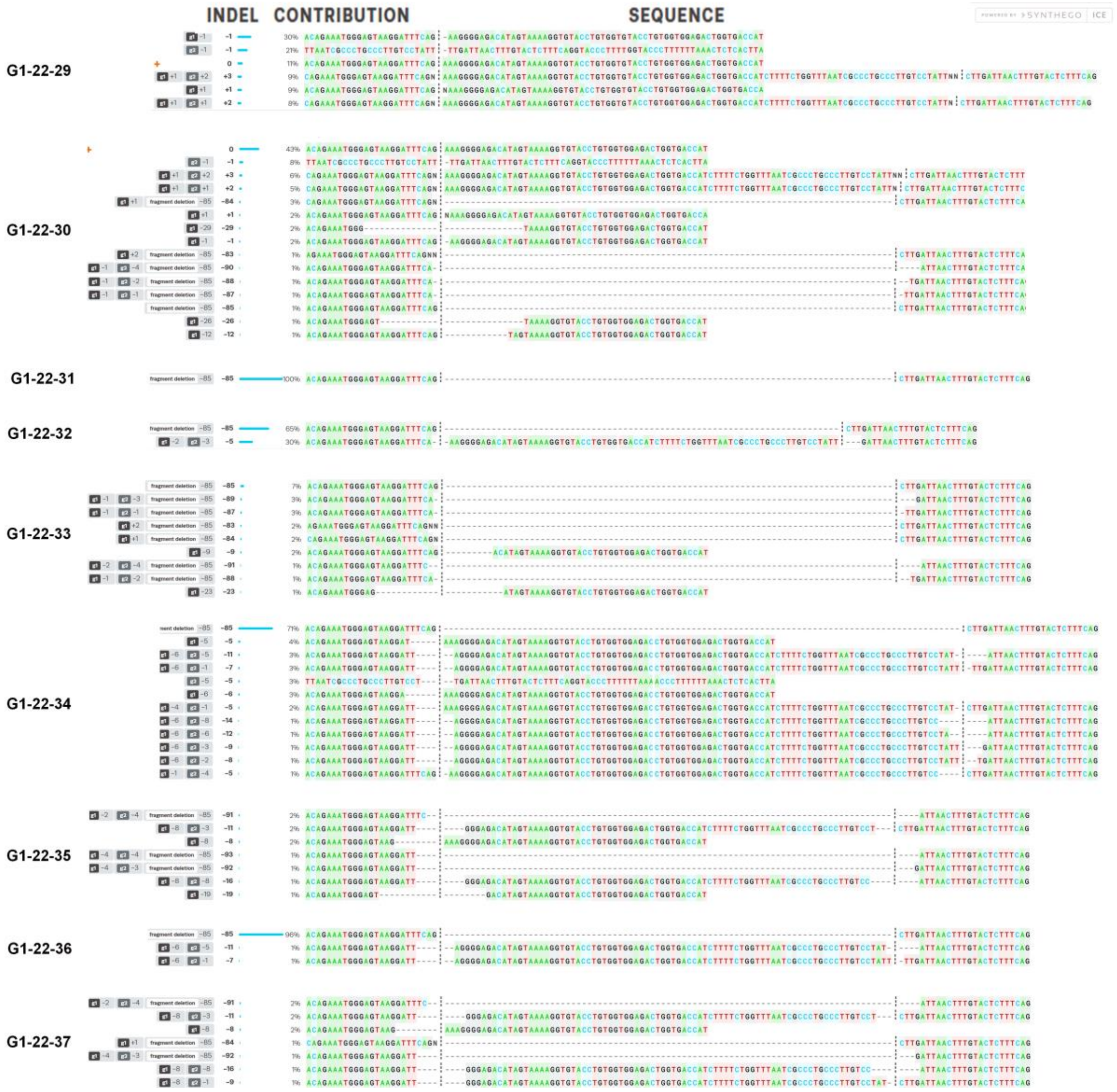

Figure S7.

CRISPR/Cas9-generated *SlMlo1*-edited alleles in the G1 generation.
Sanger sequencing data of individual G1 plants were analyzed using the ICE Synthego tool. The *SlMlo1* mutant allele with indel pattern and contribution of editing efficiency indicated. The SpCas9-sgRNA cleavage site marked with dotted vertical lines and nucleotide deletions drawn with dashes.

Table S1 List of the primers used in this study.

No.	Primer Name	Sequence (5'-3')	Purpose	Product size (bp)
1	66F	TGTGGTCTCAGATTGTGATGGTTCTGGTA GTGTAAGTTTTAGAGCTAGAAATAGCAAG	*SlPelo*-Target A cloning	-
2	67F	TGTGGTCTCAGATTGGCTTATAATCTGAT AGCTGAGTTTTAGAGCTAGAAATAGCAAG	*SlPelo*-Target B cloning	-
3	114F	TGTGGTCTCAATTGCGTAGAGACTTTGTTC CTGAGTTTTAGAGCTAGAAATAGCAAG	*SlPelo*-Target C cloning	-
4	115F	TGTGGTCTCAATTGCATAGATCATCAGCT TCTTCGTTTTAGAGCTAGAAATAGCAAG	*SlPelo*-Target D cloning	-
5	160F	TGTGGTCTCAATTGGCTCCAGAAGCAGCT TCCCTCGTTTTAGAGCTAGAAATAGCAAG	*SlPelo*-Target1 cloning	-
6	161F	TGTGGTCTCAATTGGATACTTCATCTCGAC CACATGTTTTAGAGCTAGAAATAGCAAG	*SlPelo*-Target2 cloning	-
7	162F	TGTGGTCTCAATTGGATTCTTCAACTTAAT CCTTTGTTTTAGAGCTAGAAATAGCAAG	*SlPelo*-Target3 cloning	-
8	154F	TGTGGTCTCAATTGGATTCTTCCCGCGAAT ACGCAAGGGTTTTAGAGCTAGAAATAGCA AG	*SlPelo*-Target4 cloning	-
9	163F	TGTGGTCTCAATTGGCTAATTATTTTATTG CAGATGTTTTAGAGCTAGAAATAGCAAG	*SlPelo*-Target5 cloning	-
10	87F	TGTGGTCTCAATTGGGGAGTAAGGATTTC AGAAAGTTTTAGAGCTAGAAATAGCAAG	Mlo1 gRNA1-cloning	-
11	88F	TGTGGTCTCAATTGGTACAAAGTTAATCA AGAATGTTTTAGAGCTAGAAATAGCAAG	Mlo1 gRNA-2 cloning	-
12	70R	TGTGGTCTCAAGCGTAATGCCAACTTTGT AC	Universal Rev. primer for gRNA-scaffold cloning	-
13	gRNA-A-F	TGTGGTCTCAGATTGGTGATGGTTCTGGT AGTGTAAGTTTTAGAGCTAGAAATAGCAA G	*SlPelo* sgRNA-A cloning	-
14	gRNA-B-F	TGTGGTCTCAGATTGGGCTTATAATCTGA TAGCTGAGTTTTAGAGCTAGAAATAGCAA G	*SlPelo* sgRNA-B cloning	-
15	gRNA-C-F	TGTGGTCTCAGATTGGCGTAGAGACTTTG TTCCTGAGTTTTAGAGCTAGAAATAGCAA G	*SlPelo* sgRNA-C cloning	-
16	gRNA-D-F	TGTGGTCTCAGATTGGCATAGATCATCAG CTTCTTCGTTTTAGAGCTAGAAATAGCAA G	*SlPelo* sgRNA-D cloning	-
17	138F	GACGAGTACAAGGTGCCGAGCA	spCas9-F/R(partial)	922
18	139R	GGTGGTGCTCATCATAGCGCT		
19	142F	ATGTGAATCGGGACCACAC	*SlMlo1* flanking primers	816
20	144R	GGTCTGCAGCATTCTTATGAAA		

21	149F	GGTAAGCTATTGACACATTGTAT	*Pelo* flanking primers (Target A to D)	1108
22	150R	CCATGAGATTCAAAAGTCGTTC		
23	158F	GGGTCTTTGCTGATTGTTAAC	*Pelo* flanking primers (Target 1 to 5)	943
24	159R	CATATATCACCAGCTTGATAGC		
25	GAPDH-F	GATTCGGAAGAATTGGCCG	GAPDH	606
26	GAPDH-R	TCATCATACACACGGTGAC		
27	pJET1.2 F	CGACTCACTATAGGGAGAGCGGC	TA cloning	-
28	pJET1.2 R	AAGAACATCGATTTTCCATGGCAG		
29	L2-F	GGCAGGATATATTGTGGTGTAAAC	Sequencing for L2 plasmids	-
30	L2-F	GTTTACCCGCCAATATATCCTGTC		
31	qTYLCV-C1-F	GCTCGTAGAGGGTGACGAA	RT-PCR	-
32	qTYLCV-C1-R	CACAAAGTACGGGAAGCCCA		-
33	EF-1αF	GGAACTTGAGAAGGAGCCTAAG		-
34	EF-1αR	CAACACCAACAGCAACAGTCT		-
35	On-F	CCCACCCGTGTCGATTTCTA	16S rRNA specific primes for *On*	404
36	On-R	CCGCCACTATCTTTAAGAGCTG		
37	off-1-F	GGTGTTCGACTTTCACTTG	off-target analysis	549
38	off-1-R	GGTGGTTGAAATTGTTGAAAG		
39	off-2-F	CAGATGCACCAGAGTACATG		569
40	off-2-R	CTGGTGACTTCAGTCTCTG		
41	off-3-F	CCGCGTCTCCACTAAAATG		447
42	off-3-R	CCATACTGTGTAAACGTAGGG		
43	Off-4-F	GCGAAAATAAGCATGGCATAAAG		378
44	Off-4-R	CTCAAATGTTGTGGTGAGTAGTG		
45	off-5-F	CCGATGAATGGGAAGATTGA		581
46	off-5-R	TCTCATCTGACTGCGAGC		
47	Off-6-F	TGCACTTCACTAACAACCCA		358
48	Off-6-R	CTAAGCCTATGCAAATTCATTCTC		
49	Off-7-F	AGTGATGCGACAGTTTGGT		557
50	Off-7-R	CGTACGGCTCAGTGAACAC		
51	off-8-F	AAATGGGTCAGGCGTGTCA		479
52	off-8-R	AGTCGATGGTTCAGCTTCTC		
53	off-9-F	CATCCTCCAAGAATGGGTCG		475
54	off-9-R	TTGATGGCTCGGCTCTTCTT		

55	off-10-F	CGATAATTCCGTTGATCCGT		512
56	off-10-R	GGAATTTGAGAATACATTCCCTCC		
57	off-11-F	GCTTATGCACTAGGGTGGT		460
58	off-11-R	CACTTGAATGATCTGTGGGAC		
59	off-12-F	GAGGCAAGTAAAGGAGGGC		584
60	off-12-R	CAGCCGGAAAGATGAAACTGT		

Table S2. gRNAs sequences and potential off-target sites evaluated.

Gene	Target	Target sequence with PAM (5'-3')	Chromosome	Position	Direction	Mismatches	Off-target mutation	
							G1-41-40	G1-22-32
SlPelo	on-target-1	**CTCCAGAAGCAGCTTCCCTCAGG**	SL2.40ch04	3124039	+	-	NA	NA
	on-target-2	**ATACTTCATCTCGACCACATTGG**	SL2.40ch04	3123787	+	-	NA	NA
	off-target-2-1	**ATACTTCAcCTCGACCACcaTGG**	SL2.40ch04	18337804	-	3	0	0
	on-target-3	**ATTCTTCCCGCGAATACGCAAGG**	SL2.40ch04	3123483	+	-	NA	NA
	off-target-3-1	**ATTtTTCAACTccATCCTTTTGG**	SL2.40ch12	12025128	-	3	0	0
	on-target-4	**ATTCTTCAACTTAATCCTTTTGG**	SL2.40ch04	3123430	-	-	NA	NA
	off-target-4-1	**ATTtTTCtcCTTAATCCTTTTGG**	SL2.40ch02	43011559	+	3	0	0
	on-target-5	**CTAATTATTTTATTGCAGATAGG**	SL2.40ch04	3123370	-	-	NA	NA
	off-target-5-1	**tTAATTtTTTTATTGgAGATGGG**	SL2.40ch06	16572393	-	3	0	0
	off-target-5-2	**CTAATaATaTTATTGgAGATCGG**	SL2.40ch03	58511693	-	3	0	0
	off-target-5-3	**CTAAaTATTTTcTTaCAGATAGG**	SL2.40ch04	27617425	+	3	*	0
SlMlo1	on-target-6	**GGGAGTAAGGATTTCAGAAAGGG**	SL2.40ch04	38701781	-	-	NA	NA
	off-target-6-1	**GGGAGcAAGaATTTCAGcAAAGG**	SL2.40ch01	19670702	-	3	*	*
	off-target-6-2	**GGGAGcAAGaATTTCAGAAgAGG**	SL2.40ch03	4352564	+	3	*	*
	off-target-6-3	**GGGAGcAAGaATTTCAGcAAAGG**	SL2.40ch03	43975264	-	3	0	0
	on-target	**GTACAAAGTTAATCAAGAATAGG**	SL2.40ch04	38701685	+	-	NA	NA
	off-target-7-1	**GTACAgAGTTAATgAAtAATAGG**	SL2.40ch02	42572234	-	3	0	0
	off-target-7-2	**GTACAAAGTTgAcaAAGAATGGG**	SL2.40ch03	19272711	-	3	0	0
	off-target-7-3	**ATACcAAGTTAgTgAAGAATTGG**	SL2.40ch05	54920595	+	3	0	0

NA- Not applicable. * failed in Sanger sequencing due to noise.
